# AUREA: an open-source software system for accurate and user-friendly identification of relative expression molecular signatures

**DOI:** 10.1186/1471-2105-14-78

**Published:** 2013-03-05

**Authors:** John C Earls, James A Eddy, Cory C Funk, Younhee Ko, Andrew T Magis, Nathan D Price

**Affiliations:** 1 Institute for Systems Biology, Seattle, WA, USA; 2 Department of Computer Science and Engineering, University of Washington, Seattle, WA, USA; 3 Department of Bioengineering, University of Illinois, Urbana, IL, USA; 4 Center for Biophysics and Computational Biology, University of Illinois, Urbana, IL, USA; 5 Department of Computer Science, University of Illinois, Urbana, IL

## Abstract

**Background:**

Public databases such as the NCBI Gene Expression Omnibus contain extensive and exponentially increasing amounts of high-throughput data that can be applied to molecular phenotype characterization. Collectively, these data can be analyzed for such purposes as disease diagnosis or phenotype classification. One family of algorithms that has proven useful for disease classification is based on relative expression analysis and includes the Top-Scoring Pair (TSP), k-Top-Scoring Pairs (k-TSP), Top-Scoring Triplet (TST) and Differential Rank Conservation (DIRAC) algorithms. These relative expression analysis algorithms hold significant advantages for identifying interpretable molecular signatures for disease classification, and have been implemented previously on a variety of computational platforms with varying degrees of usability. To increase the user-base and maximize the utility of these methods, we developed the program AUREA (Adaptive Unified Relative Expression Analyzer)—a cross-platform tool that has a consistent application programming interface (API), an easy-to-use graphical user interface (GUI), fast running times and automated parameter discovery.

**Results:**

Herein, we describe AUREA, an efficient, cohesive, and user-friendly open-source software system that comprises a suite of methods for relative expression analysis. AUREA incorporates existing methods, while extending their capabilities and bringing uniformity to their interfaces. We demonstrate that combining these algorithms and adaptively tuning parameters on the training sets makes these algorithms more consistent in their performance and demonstrate the effectiveness of our adaptive parameter tuner by comparing accuracy across diverse datasets.

**Conclusions:**

We have integrated several relative expression analysis algorithms and provided a unified interface for their implementation while making data acquisition, parameter fixing, data merging, and results analysis ‘point-and-click’ simple. The unified interface and the adaptive parameter tuning of AUREA provide an effective framework in which to investigate the massive amounts of publically available data by both ‘*in silico*’ and ‘bench’ scientists. AUREA can be found at http://price.systemsbiology.net/AUREA/.

## Background

Relative expression analysis examines the relative ranked abundance of genes, as opposed to absolute gene expression values. The basic unit of comparison is the relative expression reversal, where the expression value between two biomolecules (e.g., genes) reverses between phenotypes. These rank comparisons can then be generalize to larger numbers of biomolecules [1]. Changes in the relative ranks of expression have been shown in several studies to accurately classify disease phenotypes, cancer subclasses, and disease outcomes based on tumor-derived RNA expression profiling [2-6]. Relative expression analysis also has tremendous potential for identification of potential diagnostic and prognostic markers, and can function as a hypothesis generator for investigating the underlying biological processes of interest.

Currently, subsets of relative expression analyses are available as Matlab functions [7,8], Perl scripts [6], and R scripts [3,9]. The limited availability of these algorithms on specific platforms restricts their usage to researchers well versed in the languages in which they were developed or familiar with the environments in which they are implemented. AUREA is the first application that provides these algorithms (TSP, k-TSP, TST, and DIRAC) in a unified framework and with a simple-to-use graphical user interface (GUI). AUREA is open source and available for all major operating systems. Furthermore, the GUI enables intuitive control over the parsing of input data, which can sometimes be a bottleneck given the lack of standard formats across gene expression studies. Finally, while the methods combined in AUREA are designed to operate with relatively few tunable parameters, the choice of settings—and even algorithm—to obtain the best classification signature presents an extensive search space for both lay and advanced users. We have addressed this problem by including a optimization-based approach to adaptively determine algorithm settings. We hope the accessibility of AUREA, combined with efficient data processing features and adaptive parameter tuning, will allow a broader range of scientists to incorporate relative expression methods into their analytical toolbox.

## Implementation

### AUREA: an overview

AUREA incorporates the relative expression analysis algorithms TSP, k-TSP, TST and DIRAC. The Top-Scoring Pair (TSP) [10] algorithm identifies the pair of genes with the maximum likelihood of being ordered consistently within each class, but differently between classes. The k-TSP [6] algorithm, by extension, uses internal cross-validation (or similar) to identify the set of ‘k’ or less TSPs (where each gene is permitted to appear in only one TSP) that exhibits the greatest collective accuracy for separating classes when combined by majority vote. The Top-Scoring Triplet (TST) [3] algorithm identifies the triplet of genes with the maximum likelihood of being ordered consistently within each class, but differently between given classes. The Differential Rank Conservation (DIRAC) [7] algorithm identifies a gene set (e.g., signalling or metabolic pathway) with maximum likelihood of showing consistent relative expression (i.e., ranking of all network genes, from highest to lowest expression) within a class, while displaying different relative expression between given classes.

Unification of methods was achieved through developing a consistent programming interface across all algorithms. This unified interface allowed us to create a single data acquisition/presentation layer that is extensible and easy to use. AUREA can process SOFT [11] and comma-separated values (CSV)-formatted data files, which are handled by parser sub-modules in the AUREA system library. Extending AUREA to new user-specified data file formats requires development of a parser for the new data format and presentation of the results of the parser to the packager module, which then presents a consistent data layer to the rest of the system. The merging of multiple data files is also handled in the packager module, via a Python implementation of Babel [12], which maps between different microarray platforms. Within the GUI, users are not required to know anything about these processes because data acquisition, parsing and merging are handled automatically.

Adaptive parameter tuning with the relative expression analysis algorithms was also enabled by the consistent programming interface. This tuning initiates a broad search of algorithm parameter space, which is guided by accuracy, running time and the relative number of iterations an algorithm has previously performed. Using these variables as heuristics allows a simple and effective automated method of finding the parameters and algorithm that best characterize the phenotype of interest.

### Using AUREA: an example workflow

**Figure 1:**
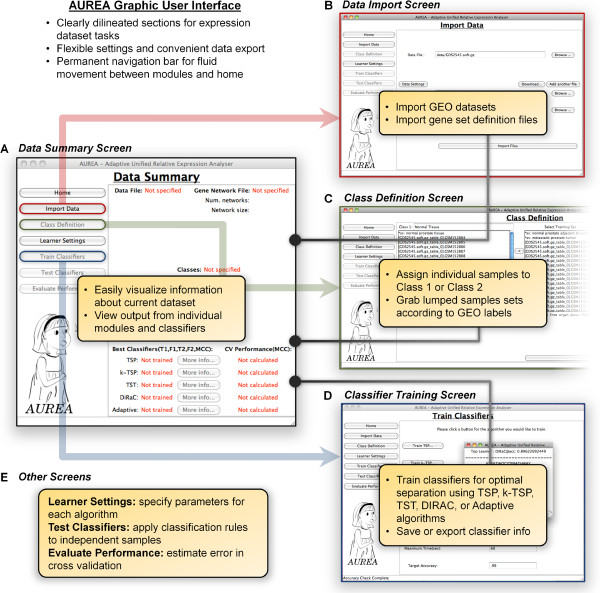
The AUREA GUI.

Prototypical usage of AUREA begins with a selected dataset of interest (containing multiple related samples of microarray expression data) from the GEO database [13]. Upon opening the AUREA GUI, the *Data Summary* screen is displayed (Figure 1A), with button links to each task or module on the left side. While AUREA allows for some tasks to be performed in a nonlinear manner, the links are ordered according to the most logical flow in a typical scenario. The first task in this example is acquiring the data set; data are imported via the *Import Data* screen (Figure 1B), which is accessible by clicking *Import Data*. At the *Import Data* screen, data can be added for analysis by either browsing the local hard drive or by entering the GEO accession in the *Download* dialog box. Entry of the GEO accession number initiates automatic retrieval of the data from GEO, which are added to the workspace. If the user intends to run either the DIRAC or Adaptive algorithm, specification of a Gene Network File to map genes onto networks is also required on this screen (*c2.biocarta.v2.5.symbols.gmt*[14] is provided by default); such files typically comprise dozens to hundreds of gene lists representing biological pathways (e.g., signaling, metabolic) or other relevant groupings. This specification can also be done at a later time. Once all data files are specified, the task is executed by clicking the *Import Files* button.

After expression data have been imported, profiles in the dataset must be partitioned into two groups (i.e., Class 1 and Class 2) prior to classifier training. These groups represent the two classes that the learning algorithms are attempting to discriminate. The *Class Definition* screen (Figure 1C) provides descriptive information and convenient functionality for completing this step. Single or multiple samples can be selected and moved to Class 1 or Class 2; samples not moved to one of the two groups will not be considered in the analysis. SOFT files from GEO have the additional benefit of grouping samples into various cohorts based on the meta-data provided. This information for individual samples can be easily viewed at the bottom of the screen, and each profile can then be moved to Class 1 or Class 2 by clicking the left or right arrow, respectively. Alternatively, selecting and moving any of the individual cohort tags automatically moves all samples within the cohort to the corresponding class, allowing for batch partitioning of the data—i.e., all samples with the ‘cancer’ subset tag can be moved to Class 1 or Class 2 with one click).

The final step is training classifiers on the defined classes. Each individual relative expression algorithm (TSP, k-TSP, TST, and DIRAC) can be run from the *Train Classifiers* screen (Figure 1D) to identify the associated signature (gene pairs, gene triples, or gene networks) that best distinguishes between the defined classes. By selecting Adaptive Training, AUREA runs through a combination of settings and algorithms, learning which appears to be most useful for the dataset (based on k-fold cross-validation accuracy). When the training times out (at a user-specified time limit) or an algorithm reaches perfect accuracy on cross-validation, AUREA returns the results of the training, reporting the accuracy and the molecular phenotype characteristics that were most discriminative from the defined classes.

At any point during the analysis, returning to the *Home* screen will give a summary report on the current state of the analysis. The user can also perform k-fold cross-validation on the dataset for each of the learning algorithms (including the Adaptive trainer) or select a subset of the data for classification (Figure 1E).

### AUREA libraries: system design

The GUI was designed to utilize the information provided by relative expression analysis. The unification of the learning algorithms was achieved through object-oriented design. Encapsulation of the learning algorithms into self-contained modules with compatible interfaces was done throughout the design of the rest of the system. While AUREA is a desktop application designed for use by people with little programming expertise, AUREA is also a software library that maintains the same functionality for the Python interpreter as is available through the GUI. This means that scripts can be written in Python to perform any of the included algorithms.

Python was chosen for this project because it is a cross-platform language that is fully object-oriented, uses a simple syntax and has a large developer community. AUREA was designed to impose a minimal number of dependencies on the user. The only external module required is the Tk/Tcl windowing module, allowing cross-platform GUIs. Scripts can be run from any standard Python installation, version 2.6 or higher.

The AUREA libraries are broken up into a set of modules: the GUI, packager, learner, adaptive and parser modules. The GUI module handles the graphical interface to the AUREA libraries. The learner module contains the relative expression algorithms. The adaptive module provides the adaptive learning features in AUREA. The parser and packager modules are of particular interest to those who wish to extend AUREA to their problem domains; the packager module contains objects that take parser objects, transform those objects into data tables, and then merge those data tables to create inputs for the learning algorithms. As such, extending the functionality of AUREA to an unsupported data format only requires a new parser to provide a mapping of the parse to the packager module.

Another extensible aspect of the AUREA libraries is the learner module, which allows for the addition of a new learning algorithm using the interface of the existing relative expression analysis algorithms. By implementing *train*, *classify* and *crossValidate* methods for the new learning algorithm, AUREA can utilize the new method as if it were one of the stock algorithms, greatly decreasing development time by removing the need to develop an entirely new framework. It is not necessary to develop a new learning algorithm in C or another lower level language, as was done with the current relative expression analysis algorithms, but it is recommended, since Python, as an interpreted language, is much slower than a compiled language like C/C++. In accordance with this point, AUREA uses Python where performance is I/O bounded, i.e., in the parsers and graphical interfaces, and as a wrapper around the optimized C/C++ code on the computationally intensive relative expression algorithms.

The API documentation is available [15], and all code is provided under the GPL v. 3 Affero license. Developers can use the source code to create their own adaptations of AUREA, although we encourage them to contribute their changes back to the main project to simplify ease of use in the community.

## Results and discussion

Using AUREA, we analyzed datasets representing diverse disease phenotypes, clinical outcomes and tissue types. These analyses were carried out to demonstrate the performance of the adaptive parameter tuning module; however, they also demonstrate the flexibility of the methods AUREA employs, and show that AUREA can be utilized by researchers as a hypothesis generator in crafting directed inquiries into the molecular characteristics of the phenotypes being studied.

### Adaptive parameter tuning

Each algorithm used in relative expression analysis relies on parameters—albeit relatively few compared to those needed for most classification algorithms—that affect accuracy and running time. Finding the best set of parameters for a given dataset can be a time-consuming task. For example, TSP, TST and k-TSP can all operate on a subset of genes for analysis based on the differential expression of the presented gene set (the Wilcoxon signed-rank test is used to choose the most differentially expressed genes between the defined classes). Aside from the dramatic effect that this feature selection step can have on the computational complexity of the algorithms (TST is computationally infeasible over a complete transcriptome on standard hardware, though reachable on GPUs [8]), very few conditions result in a complete change in genomic expression; examining too large a set of genes can therefore result in over-fitting, and lead to spurious results. The performance of DIRAC, on the other hand, is sensitive to the minimum size of the networks examined and the number of networks used for classification. The adaptive parameter-tuning tool automates the process of finding both the relative expression algorithm and associated settings—the Algorithm-Parameter Configurations (APCs)—that most accurately characterize the selected dataset.

The adaptive module computes the estimated running time for each algorithm based on a user-defined range of parameter values; it generates a score for each possible configuration using the estimated running time and the previous accuracy of the algorithm on the data set. In short, an algorithm’s likelihood of running is inversely proportional to the estimated running time, inversely proportional to the number of times it has run, and proportional to how well it scored the previous times it ran. The score is defined as follows:

(1) $$\delta \left(x\right)=\frac{t\left(x\right)}{\prod_{y\in X}\alpha \left(y\right)}$$

where *t*(*x*) is the estimated running time of the algorithm, *X* is the set of all the previously run learning algorithms of the same type as *x* and α(*y*) is the linear mapping of the Matthews Correlation Coefficient (MCC)—our selected metric for classification accuracy, which ranges from −1 (total disagreement between prediction and observation) and 1 (perfect agreement)—of *y* (prior iterations of the algorithm) to (0,1). The adaptive module then runs the APC with the smallest δ. This process can be run exhaustively, but generally is restricted to run until some time limit is reached, or a sufficient accuracy in the training set is achieved.

**Figure 2:**
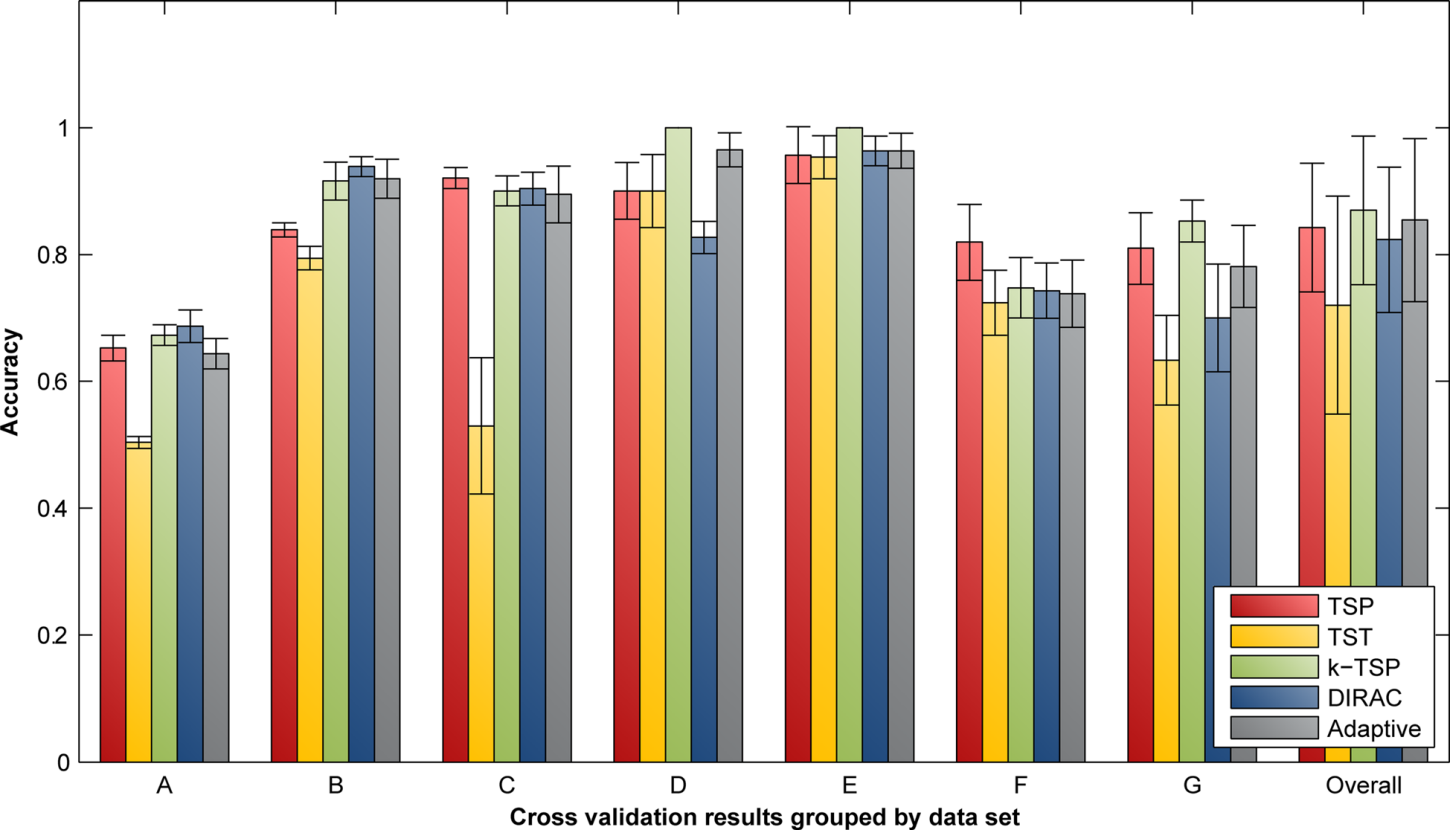
**A comparison of the cross-validation accuracies.** The relative expression analyzers were averaged over 10 runs of 10-fold cross-validation, with the error bars showing the standard deviation between the individual runs. All datasets are from GEO [13]. (**A**) GDS2545 normal prostate adjacent to tumor v. primary prostate tumor, (**B**) GDS2545 primary prostate tumor v. normal prostate, (**C**) GDS1209 normal v. sarcoma, (**D**) GDS1210 normal v. cancer, (**E**) GDS1269 non-smoker control v. smoker, (**F**) GDS1330 normal v. Crohn’s disease, (**G**) GDS1330 normal v. ulcerative colitis.

Our tests show the variability of accuracy between different phenotype studies and demonstrate that while the adaptive module does not always achieve the highest accuracy, it does generally have an accuracy level comparable to the best possible, making it a good default choice for exploring a dataset (see Figure 2).

### Targeted users

AUREA is targeted to a diverse community of biological researchers and can be leveraged to improve analysis, streamline development cycles and guide human intuition. AUREA simplifies the process of data analysis and new tool development, whether the goal involves investigation of new phenotypes or applying a new algorithm. A biologist looking to quickly view a new dataset or a computer scientist wanting to test a new classification scheme can quickly and easily leverage AUREA.

AUREA (through the GUI) is designed to remove the technical barriers to using relative expression analysis by simplifying and streamlining the discovery process. Without the need of stringing together multiple command line tools across languages and platforms, each with its own learning curve, biologists can instead focus on the biological, as opposed to technical, problems underlying molecular phenotype characterization.

The modular design of the AUREA software libraries presents many opportunities for computational biologists to integrate or extend the capabilities of relative expression algorithms. We have successfully used AUREA on clusters and desktops, integrating it with databases and, in a dozen short lines of code, performing more complex molecular phenotype characterization on a diverse range of projects.

For computer scientists, AUREA presents a platform on which to create and run their own classification algorithms, without the need to develop supporting software for data acquisition and user interaction.

### Comparison with existing implementations

Current implementations of relative expression analysis methods include the tspair [9] package, which is included in the Bioconductor suite of R software; RXA [3], also in R, which includes TSP and TST; Tan et al.’s k-TSP[6] PERL package and the original DIRAC [7] implementation, available as a MATLAB script (Table 1). None of these implementations provide integrated data parsing or pre-processing capabilities, although it should be noted that each environment does have a rich set of tools for doing so. Furthermore, none of these programs provide a graphical interface, which restricts their use to analysts familiar with the programming environment. AUREA is the only implementation to automatically examine the parameter space and assist the analyst in determining the best settings for their data set; it is also the only tool that includes all four of these methods in one package. It should be noted that each of these implementations has unique advantages and features such as the tspair package’s significance testing routine.

**Table 1:** Comparison of relative expression analysis software

**Implementation**	**Lang**	**Interface**	**TSP**	**TST**	**k-TSP**	**DIRAC**	**Data parsing**
tspair [8]	R	R console	X				
RXA [2]	R	R console	X	X			
k-TSP [5]	PERL	Command Line			X		
DIRAC [6]	MATLAB	MATLAB console				X	
AUREA	Python	GUI	X	X	X	X	X

## Conclusions

AUREA is an easy-to-use, open-source, cross-platform system that unites several relative expression analysis algorithms in a framework that enables application by a broad range of users. Through minimal system requirements and simplicity of interface and configuration, these tools can be applied far more casually and broadly than previously possible. As computational tools become more powerful and easier to understand through reduced technological overhead, they will become more accessible to non-computational scientists, who can use them to fully harness actionable biological meaning and understanding.

### Source code

All of the source code and documentation is available on the Price Lab Website (http://price.systemsbiology.net/AUREA/).

## Availability and requirements

• **Project Name:** AUREA (Adaptive Unified Relative Expression Analyzer)

• **Project home page:**http://price.systemsbiology.net/AUREA/

• **Operating System(s):** Windows, OS X, Linux

• **Programming Language(s):** Python, C/C++, SWiG 1.3.36

• **Other Requirements:** Python 2.6.x or 2.7.x, Tkinter package (standard in most python packages)

• **License:** GNU Affero General Public License v.3

## Competing interests

The authors declare that they have no competing interests.

## Authors’ contributions

JCE provided the primary design and development of the system. NDP and JAE conceived and directed the project. JAE contributed to the development of the DIRAC module. ATM contributed to the development of TSP and TST modules. YK contributed to the development of the Adaptive Module, the Wilcoxon Module and the k-TSP module. JCE, JAE, ATM and YK performed software testing and debugging. All persons contributed to the writing and editing of the manuscript. All authors read and approved the final manuscript.
